# Identification of Potential Roles of Cathepsin B-like in the Response to Alkali Treatment in *Macrobrachium nipponense*

**DOI:** 10.3390/ijms26073361

**Published:** 2025-04-03

**Authors:** Mingjia Xu, Wenyi Zhang, Yiwei Xiong, Hongtuo Fu, Hui Qiao, Sufei Jiang, Shubo Jin

**Affiliations:** 1 Wuxi Fisheries College, Nanjing Agricultural University, Wuxi 214081, China; xumingjia8562@163.com (M.X.); fuht@ffrc.cn (H.F.); jiangsf@ffrc.cn (S.J.); 2 Key Laboratory of Freshwater Fisheries and Germplasm Resources Utilization, Ministry of Agriculture and Rural Affairs, Freshwater Fisheries Research Center, Chinese Academy of Fishery Sciences, Wuxi 214081, China; zhangwy@ffrc.cn (W.Z.); xiongyw@ffrc.cn (Y.X.)

**Keywords:** *Macrobrachium nipponense*, alkali treatment, cathepsin B-like, qPCR, RNAi

## Abstract

Cathepsin B is a member of the cysteine protease family and plays an important role in the innate immunity of aquatic invertebrates. A previous study identified that Cathepsin B-like (*CTSB-l*) may be involved in the response of alkali treatment in *Macrobrachium nipponense*. The present study aims to identify the potential regulatory roles of *CTSB-l* in the response of alkali treatment in *M. nipponense* through performing the quantitative real-time PCR analysis (qPCR), in situ hybridization (ISH) analysis, and RNA interference (RNAi) analysis. The full length of the *MnCTSB-l* cDNA was 1272 bp with an open reading frame of 987 bp, encoding 328 amino acids. Phylogenetic tree analysis indicated that the amino acid sequence of *MnCTSB-l* is highly homologous to those of crustacean cathepsin B-like. qPCR analysis showed that *MnCTSB-l* mRNA is expressed in all tested tissues with the highest level of expression in hepatopancreas in both male and female prawns. The expressions of *MnCTSB-l* were significantly stimulated in gills under the alkali concentration of both 5 mmol/L and 10 mmol/L, predicting that this gene may be involved in the response of alkali treatment in *M. nipponense*, which was consistent with the previous study. ISH showed that *MnCTSB-l* signals were mainly observed in the hemolymph vessels and membranes of gills, as well as in the basement membranes of hepatopancreas, in both male and female prawns. RNAi analysis revealed that the injection of double-stranded RNA of *CTSB* (*dsCTSB*) resulted in a significant decrease in *MnCTSB-l* expressions. In addition, prawn cumulative mortality was significantly higher in the *dsCTSB*-injected group, compared to that of *dsGFP*-injected group, under alkali treatments of both 5 mmol/L and 10 mmol/L, indicating *CTSB-l* plays an essential role in regulating alkalinity acclimation in *M. nipponense*. The present study identifies the regulatory functions of *CTSB-l* in the response of alkali treatment in *M. nipponense*, promoting the survival rate and aquaculture of this species in a water environment with high alkalinity.

## 1. Introduction

The oriental river prawn (*Macrobrachium nipponense*) is a freshwater prawn with high economic and nutritional value, widely distributed in China, Japan, Vietnam, and other Asian countries [1]. The annual production of *M. nipponense* in China reached 226,392 tons in 2023, accounting for 5.10% of the total freshwater prawn production, with an economic value of more than 3 billion dollars. The main aquaculture regions for *M. nipponense* are located in the southeastern provinces of China, including Jiangsu province, Anhui province, Zhejiang province, and Jiangxi province, while the production in northwestern regions of China is limited [2]. A possible reason for this is that the water resources in the northwestern part of China are predominantly saline–alkali water, which have negative effects on the normal reproduction and growth of aquatic animals. A previous study determined that the 96 h *LC*_50_ value for alkalinity tolerance is 14.42 mmol/L in juvenile Taihu No2 (a genetically selected new variety of *M. nipponense*) with a safe alkalinity value of 4.71 mmol/L [3]. *M. nipponense* shows weak adaptive abilities to alkali treatment and is unsuitable for culture in water with high saline–alkali concentrations, resulting in low production in these regions [4]. Thus, it is urgently necessary to investigate the mechanism of alkali tolerance to facilitate genetic improvement in this species and develop a new variety with enhanced resistance to alkali stress.

Previous transcriptome profiling analysis was conducted in the gills of *M. nipponense* after an alkali treatment for 96 h under alkali concentrations of 4 mmol/L, 8 mmol/L, and 12 mmol/L, aiming to identify key metabolic pathways and genes involved in alkali stress responses in this species [5]. A Kyoto Encyclopedia of Genes and Genomes (KEGG) analysis revealed that the lysosomal pathway is the main enriched metabolic pathway of differentially expressed genes (DEGs). The mRNA expression levels of DEGs in this pathway progressively increased with rising alkali concentrations, indicating that both the pathway and its associated genes play essential roles in alkali adaptation in *M. nipponense*. Lysosomes are eukaryotic organelles primarily responsible for the degradation of biological macromolecules and play an important role in the stability of the internal environment of organisms [6,7]. Additionally, lysosomes are critical regulators of diverse biological processes, including intracellular transport, signaling, lipid metabolism, nutrient sensing, and immune responses [8,9,10].

The expression of Cathepsin B-like (*CTSB-l*) was significantly upregulated in *M. nipponense* following alkali treatment and was enriched in the lysosomal pathway, suggesting its role as a candidate gene responding to alkali stress in this species. Cathepsin B, a lysosomal cysteine protease belonging to the papain superfamily, is molecularly characterized by a Cys-His double amino acid group in its active center [11,12]. It is synthesized as an inactive pre-proenzyme and activates itself to become a mature form by autoproteolysis in an acidic environment [13,14]. Unlike other cathepsins, cathepsin B contains an occluding loop of 20 residues, enabling both endopeptidase and exopeptidase activity [12,15]. Initially identified in rats [16], cathepsin B has been extensively studied in aquatic species and appears pivotal in environmental stress responses. For example, its expression significantly increased in *Exopalaemon carinicauda* gills after 36 h of alkali exposure [17], whereas *Nile tilapia* exhibited downregulation following 30 days alkali treatment [18]. Similarly, pesticide exposure-induced elevated cathepsin B expression in grass shrimp [19], and nitrite stress triggered increased mRNA levels in *Litopenaeus vannamei* [20].

In this study, we aim to analyze the potential functions of *CTSB-l* in the process of alkali response in *M. nipponense* through analyzing the expressions in different tissues of both male and female prawns and the expression changes in hepatopancreas and gills after the alkali treatment by qPCR. Additionally, the cumulative mortality rate was measured in an alkali environment after the injection of *dsCTSB* and *dsGFP*. The present study provided valuable evidence for the analysis of alkali tolerance in *M. nipponense* and promoted the genetic improvement of alkali tolerance in *M. nipponense*.

## 2. Results

### 2.1. Sequence Analysis

**Figure 1:**
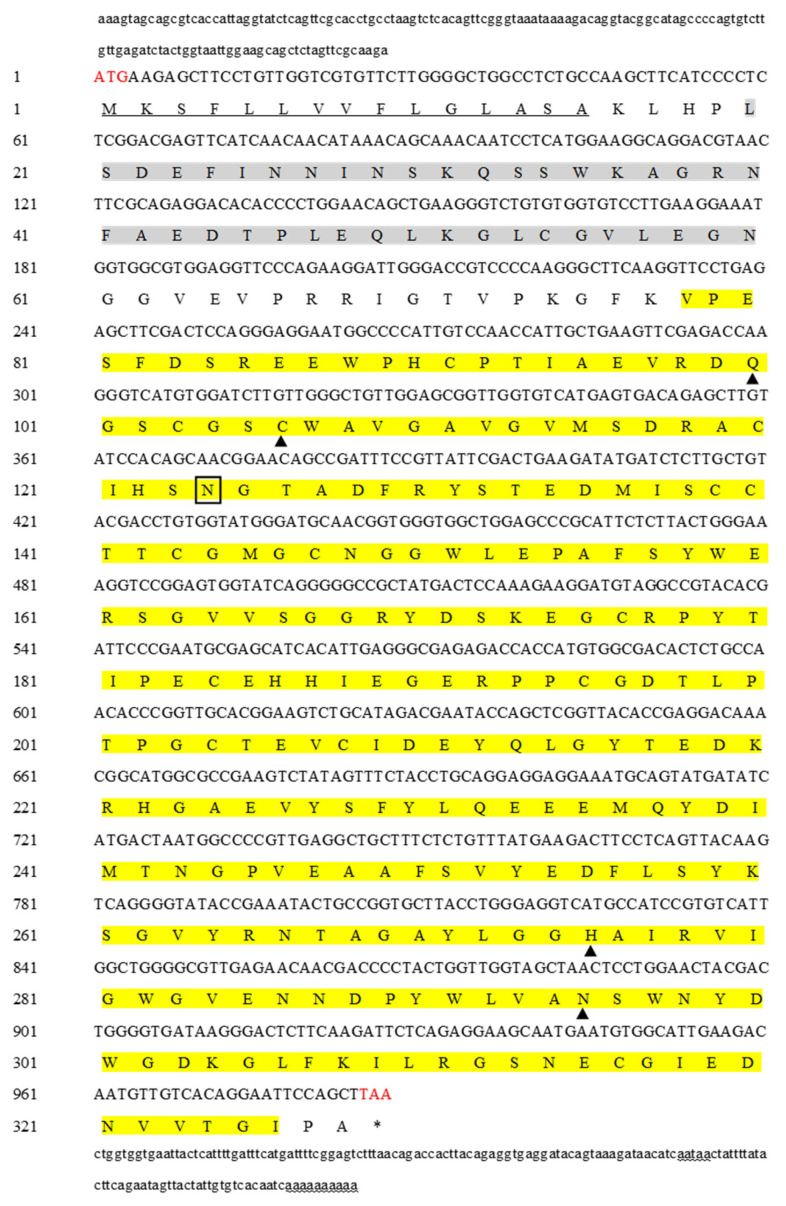
cDNA sequence and predicted amino acid sequence of *MnCTSB-l.* The start and stop codons are given in red. In the predicted amino acid sequence, the signal peptide sequence is underlined, the propeptide-C1 region is shaded, and the peptidase-C1 domain is highlighted. The four active sites are indicated with triangles. The N-glycosylation site is indicated by a box. The polyadenylation tail signal and poly(A) are indicated by wavy lines. * indicates a stop codon that does not code for an amino acid.

The full-length of the *MnCTSB-l* cDNA sequence was 1272 bp, including an ORF of 987 bp, encoding 328 amino acids (Figure 1). The predicted molecular weight and theoretical isoelectric point of the *MnCTSB-l* protein were 36.15 kDa and 4.76, respectively. The predicted *MnCTSB-l* protein consisted of a signal peptide of 15 amino acid residues, propeptide-C1 (residues 20–60), and a peptidase-C1 domain (residues 78–326). The peptidase-C1 domain contained four active site residues (Gln^100^, Cys^106^, His^275^, Asn^295^) and a potential N-glycosylation site (Asn^124^).

### 2.2. Multiple Sequence Alignment and Phylogenetic Tree Analysis

**Figure 2:**
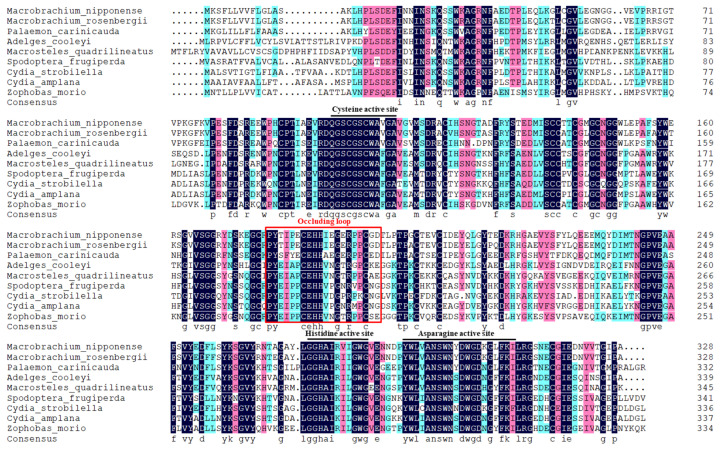
Multiple sequence comparison of cathepsin B-like from *M. nipponense* and other species. The three conserved cysteine peptidase active sites are marked with black lines. The occluding loop is boxed in red. Black area indicates identical sequences and pink area indicates conserved variants.

**Figure 3:**
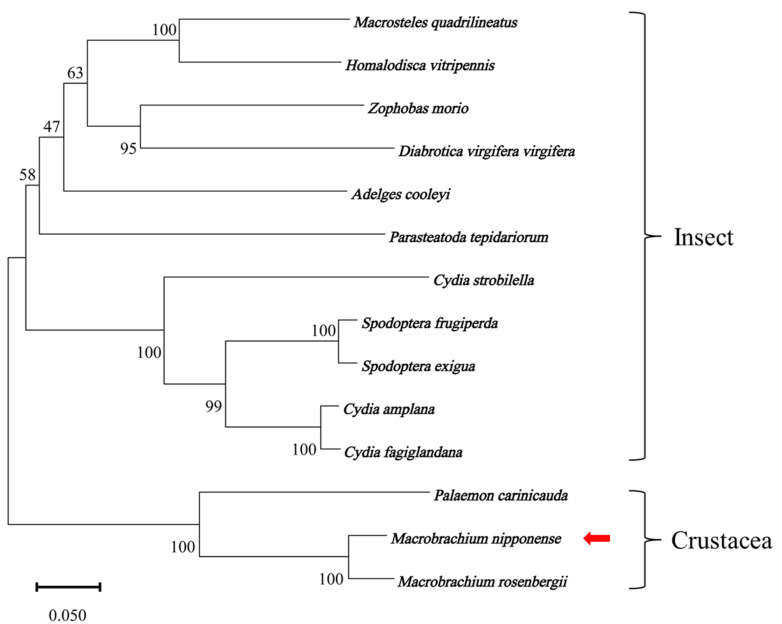
Phylogenetic tree of cathepsin B-like. *Macrobrachium nipponense* is marked with a red arrow.

Multiple sequence alignment (Figure 2) demonstrated that the identities of the amino acids of *MnCTSB-l* with those of the other identified species ranged from 54.74% to 93.60%. The *MnCTSB-l* amino acid sequence showed the highest identity with that of *Macrobrachium rosenbergii*, followed by *Palaemon carinicauda* (71.65%), while the lowest identity was identified with *Cydia amplana*. The neighbor-joining phylogenetic tree of *CTSB-l* amino acid sequences (Figure 3) delineated a conserved crustacean clade comprising *M. nipponense*, *Macrobrachium rosenbergii*, and *Palaemon carinicauda*, with insects occupying divergent evolutionary branches. *MnCTSB-l* exhibited closest evolutionary proximity to *Macrobrachium rosenbergii CTSB-l*.

### 2.3. Expression Pattern of MnCTSB-l in Tissues of M. nipponense

**Figure 4:**
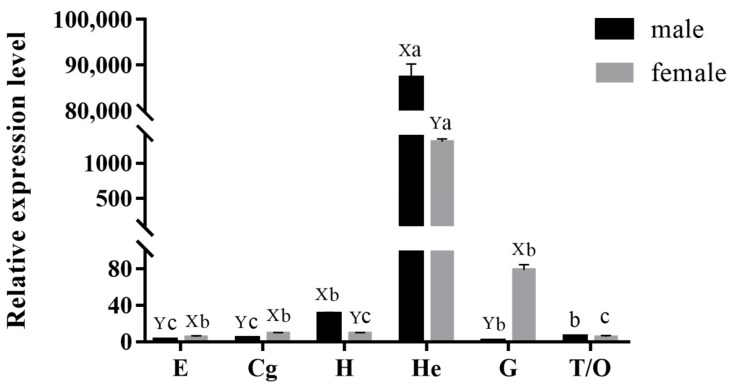
Expressions of *MnCTSB-l* in different tissues of male and female prawns. Different capital letters in the figure indicate significant differences in expression between male and female prawns in the same tissues. Different lowercase letters in the figure indicate significant differences in expression among different tissues from both sexes. E, eyestalk; Cg, cerebral ganglion; H, heart; He, hepatopancreas; G, gill; T, testis; O, ovary.

The qPCR results showed that *MnCTSB-l* mRNA was expressed in all of the tested tissues (Figure 4). The *MnCTSB-l* mRNA expressions exhibited the highest level in the hepatopancreas of both female and male prawns and showed significant differences to the other tissues (*p* < 0.05). Among all of the tested tissues, the gills of male prawns showed the lowest expression. The *MnCTSB-l* mRNA expressions in hearts and hepatopancreases of male prawns were significantly higher than those of female prawns, whereas the opposite expression patterns were found in the eyestalks, cerebral ganglions, and gills (*p* < 0.05). There were no significant differences in the mRNA expression of *MnCTSB-l* between male and female prawns in the gonads (*p* > 0.05).

### 2.4. Expression Analysis of MnCTSB-l in Hepatopancreas and Gills of M. nipponense Under Alkali Stress Conditions

**Figure 5:**
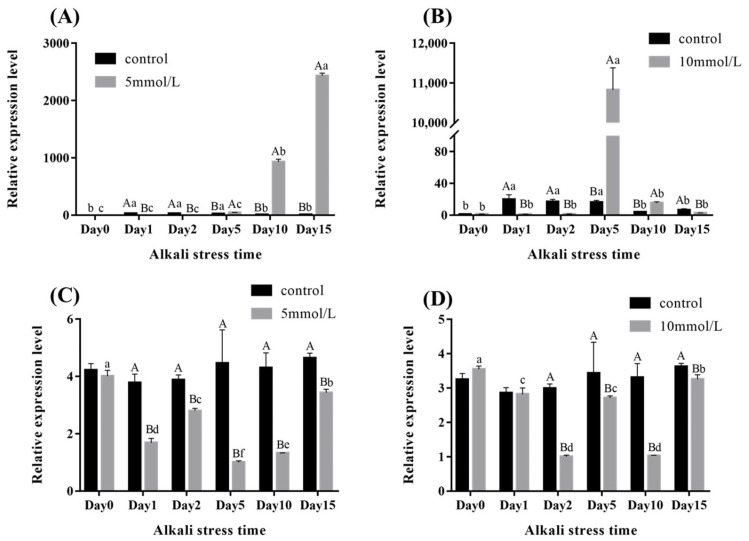
Expressions of *MnCTSB-l* in hepatopancreases and gills at different alkali concentrations. Different capital letters in the figure indicate significant differences in the expression of *MnCTSB-l* between the alkali treatment group and control group on the same days of stress. Different lowercase letters in the figure indicate significant differences in the expression of *MnCTSB-l* under different days of stress at the same alkalinity. (**A**,**B**): Expression of *MnCTSB-l* in the gills; (**C**,**D**): expression of *MnCTSB-l* in the hepatopancreases.

The expressions of *MnCTSB-l* in hepatopancreases and gills after the alkali treatment of 5 mmol/L and 10 mmol/L were shown in Figure 5. The alkali treatment stimulated the *MnCTSB-l* expressions in gills at both 5 mmol/L and 10 mmol/L. The *MnCTSB-l* expressions gradually increased with the increase in treatment time in gills under the alkali concentration of 5 mmol/L (*p* < 0.05). However, the *MnCTSB-l* expressions gradually increased and reached a peak on day 5 under alkali concentrations of 10 mmol/L (*p* < 0.05), and the expressions then decreased to below normal levels (*p* < 0.05). The expressions of *MnCTSB-l* in the hepatopancreas showed similar expression patterns under alkali concentrations of 5 mmol/L and 10 mmol/L, i.e., the expressions decreased (*p* < 0.05).

### 2.5. Localization of MnCTSB-l in the Hepatopancreases and Gills

**Figure 6:**
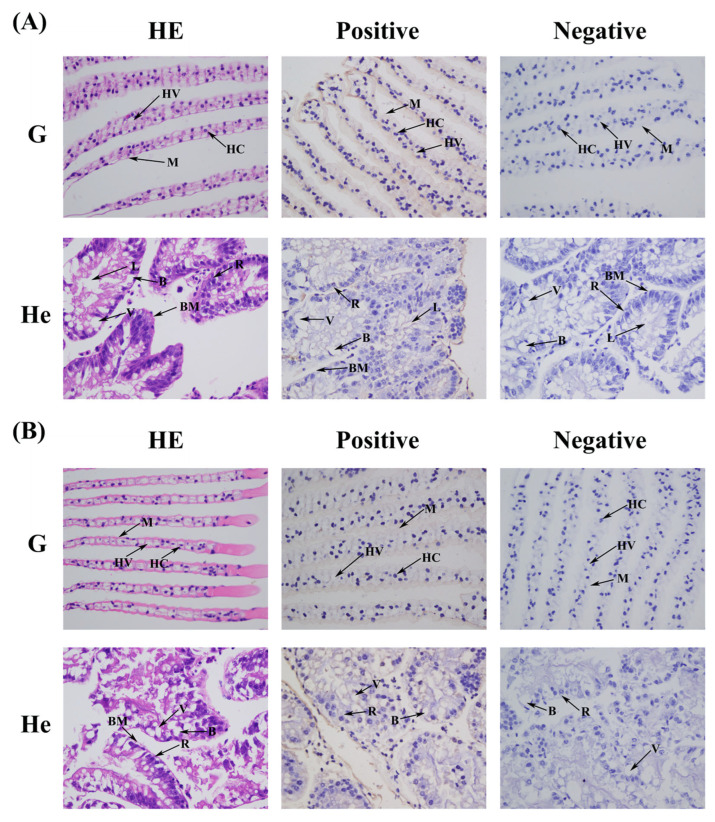
The location of *MnCTSB-l* in the hepatopancreas and gills detected by in situ hybridization. HE: Hematoxylin and Eosin staining. Positive: anti-sense probe. Negative: sense probe. (**A**) Male. (**B**) Female. B: secretory cells of type B; BM: basement membrane; L: lumen; R: storage cells of type R; V: vacuoles; HC: hemocytes; HV: hemolymph vessel; M: membrane. Scale bars = 20 μm.

ISH was used to detect the location of *MnCTSB-l* mRNA in hepatopancreas and gill tissues of both male and female prawns (Figure 6). *MnCTSB-l* signals were observed in the hepatopancreases and gills of male and female prawns, which were mainly distributed in hemolymph vessels and membranes of gills and in the basement membranes of hepatopancreases. No signal was observed in the negative control.

### 2.6. RNAi

**Figure 7:**
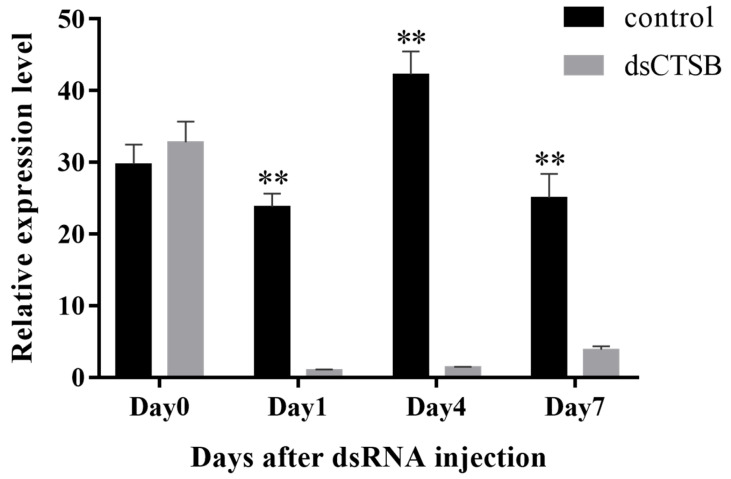
Expression level of *MnCTSB-l* in hepatopancreas after dsRNA injection. ** indicates significant differences (*p* < 0.01) in *MnCTSB-l* expression between *dsGFP*-injected group and *dsCTSB*-injected group.

The efficiency of synthesized *dsCTSB* was verified by qPCR after 7 days of *dsCTSB* injection (Figure 7). The qPCR analysis revealed that the *MnCTSB-l* expressions in the *dsCTSB*-injected groups at days 1, 4, and 7 after injection were significantly decreased by 95.79%, 96,65%, and 84.53%, respectively, compared to those of the *dsGFP*-injected group (*p* < 0.05).

**Figure 8:**
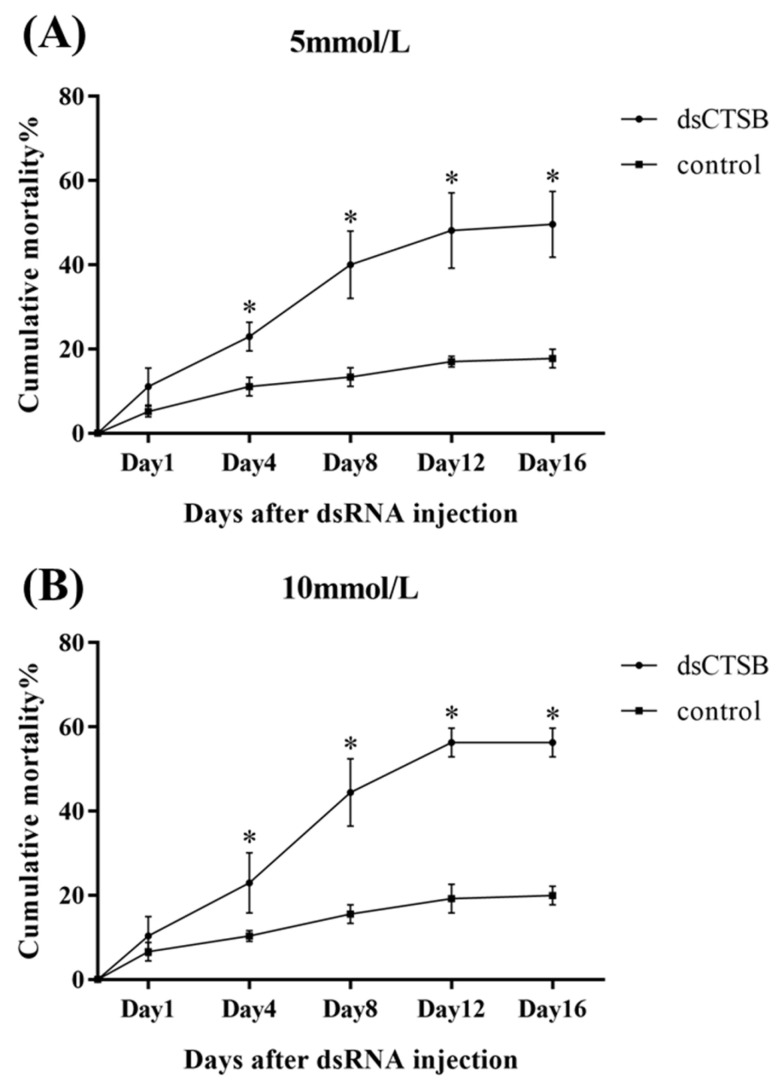
Cumulative mortality rate of prawns after dsRNA injection under alkali concentrations of (**A**) 5 mmol/L and (**B**) 10 mmol/L. * indicates significant differences (*p* < 0.05) in cumulative mortality rate between *dsGFP*-injected group and *dsCTSB*-injected group.

In the long-term interference experiment (Figure 8), the cumulative mortality rates in the *dsCTSB*-injected group under alkali concentrations of both 5 mmol/L and 10 mmol/L were over 50% after 16 days of treatment, whereas the cumulative mortality rates in the *dsGFP*-injected group were lower than 20%. The cumulative mortality rates in the *dsCTSB*-injected group were significantly higher than those in the *dsGFP*-injected group under the alkali concentrations of 5 mmol/L and 10 mmol/L after 4, 8, 12, and 16 days of treatment (*p* < 0.05).

## 3. Discussion

Cathepsin B is involved in multiple physiological processes, including inflammatory responses [21], protein degradation [15], and apoptosis [22]. Our previous study predicted that *CTSB-l* participates in alkali stress adaptation in *M. nipponense*. Therefore, the present study further investigates the potential role of *MnCTSB-l* in alkali tolerance. The full length of the *MnCTSB-l* cDNA sequence was 1272 bp, encoding 328 amino acids. The predicted structure featured a 15-residue signal peptide, a 41-residue propeptide-C1 domain, and a 249-residue mature peptidase-C1 domain, consistent with conserved cathepsin B characteristics [23,24]. Three highly conserved active sites of cysteine, histidine, and asparagine were also identified in the predicted amino acid sequences of *MnCTSB-l* (Cys^106^, His^275^, Asn^295^), which were all contained in papain-like cysteine proteases [25]. Cys^106^ and His^275^ formed the catalytic dyad (ion pair), whereas Asn^295^ and His^275^ formed the imidazole ring, both critical for enzymatic activity [24,26]. In addition, Gln^100^ contributed to oxyanion hole formation [27], and an N-glycosylation site (Asn^124^) within the mature peptidase-C1 domain was implicated in developmental regulation [28]. The occluding loop consisting of 20 amino acid residues was found in *MnCTSB-l*, which was a special structural characterization of cathepsin B [29]. Two histidine residues (His^187^ and His^188^) were observed in the occluding loop, which were essential for exopeptidase activity [30]. Multiple sequence alignment showed more than 50% identity between the amino acid sequence of *MnCTSB-l* and those of the other crustaceans and insects. Phylogenic tree analysis revealed that *MnCTSB-l* amino acid sequence had the closest evolutionary distance to crustaceans and had a significant evolutionary distance to insects. This is consistent with previous studies that revealed that *M. nipponense* has the closest evolutionary relationship with crustaceans [31,32]. Therefore, it can be inferred that *MnCTSB-l* is conserved in biological evolution.

Cathepsin B is expressed in the tissues of almost all organisms and plays an important role in many biological processes [33]. The expression level of *FcCTSB* mRNA was highest in the hepatopancreas of *Fenneropenaeus chinensis*, which was 1.6 times higher than that in the gills [34]. In *Procambarus clarkii*, *PcCTSB* mRNA was universally expressed in all tissues, with the highest expression in the hepatopancreases and abundant expression in the blood cells and gills [35]. A similar conclusion was reported for *Cristaria plicata* [29]. In this study, *MnCTSB-l* mRNA was detected in all examined tissues of *M. nipponense*, indicating its multifunctional biological roles. Furthermore, the highest expressions were observed in the hepatopancreas of both male and female prawns, which is consistent with the results of the abovementioned studies. As the primary site for protein hydrolysis, apoptosis, and immune regulation in crustaceans, the hepatopancreas serves as a key tissue for monitoring immune-related gene expression [36,37]. The high expression of *MnCTSB-l* in this immune-related tissue suggests that *MnCTSB-l* may play an important role in the immune response to the alkalinity acclimation in this species. Cellular localization analysis via ISH confirmed *MnCTSB-l* mRNA signals in hemolymph vessels and the membranes of gills and in the basement membranes of the hepatopancreases of both sexes, further suggesting that *MnCTSB-l* is involved in the immune response. This constitutes the first report of an *MnCTSB-l* investigation in this species using ISH.

Crustaceans lack adaptive immunity, relying solely on innate defense mechanisms against environmental stressors or pathogen challenges [38]. Immune defenses of organisms are activated during environmental toxic stress, and cell death/apoptosis is triggered to defend against the stress [39]. It has been reported that alkali stress can induce apoptosis and immune response in aquatic animals. For example, apoptosis and immune response occurred in gill cells of crucian carp under high alkaline stress and led to a disturbed lipid metabolism [40]. Similarly, Pacific white shrimp (*Litopenaeus vannamei*) exposed to pH 5.6 (HCl) and pH 9.3 (Na_2_CO_3_/NaHCO_3_) developed oxidative damage and apoptosis in hemocytes and hepatopancreatic cells [41]. Cathepsin B critically regulates immune cell apoptosis and activates organismal immune responses through multiple pathways [42]. Differentially expressed proteins in the gills of *Exopalaemon carinicauda* were identified under different stress times with the same alkali concentration, and cathepsin B and cathepsin L were significantly upregulated at 0 vs. 36 h [17]. In addition, the expression of cathepsin B, cathepsin L, and cathepsin Z in the gill tissues of crucian carp were significantly increased under saline–alkaline stress [43]. In the present study, the expression level of *MnCTSB-l* in gills showed an increasing trend under alkali stress at concentrations of both 5 mmol/L and 10 mmol/L, indicating that *MnCTSB-l* is involved in immune responses under alkali stress conditions. However, the expression of *MnCTSB-l* in gills decreased to a level below normal after 5 days of alkali treatment at a concentration of 10 mmol/L. A reasonable explanation for this is that the gill tissues are directly exposed to alkaline water, and the prolonged high alkalinity stress results in the impaired immune defense mechanisms of gill tissues [44]. The transcriptomic analysis of the hepatopancreases of *Exopalaemon carinicauda* exposed to carbonate (NaHCO_3_) validated the genes in the transcriptome, which showed that the expression of cathepsin B gene increased significantly under alkalinity stress conditions [45]. However, the alkali treatment did not stimulate the *MnCTSB-l* expression in the hepatopancreases of *M. nipponense*, suggesting that gills are the more important organs in terms of responses to the alkali in this organism. This is consistent with the previous studies which demonstrated that more differentially expressed genes were identified in the gills than in the hepatopancreases after the alkali treatment in *M. nipponense* [4,5].

RNAi has been extensively applied for the functional analysis of genes in *M. nippoennse*, including hypoxia-tolerance genes [46], ovarian development and molting genes [47], and reproductive regulation genes [48]. To the best of our knowledge, this study represents the first time RNAi has been used to investigate the potential functions of *CTSB-l* in the response to alkali treatment. In this study, *MnCTSB-l* expressions were significantly lower in *dsCTSB*-injected prawns than in *dsGFP*-injected prawns, indicating the synthesized *dsCTSB* can efficiently knock down the expressions of *CTSB-l* in *M. nipponense*. Furthermore, the mortality rates in the *dsCTSB*-injected group were significantly higher than those of the *dsGFP*-injected group under alkali concentrations of both 5 mmol/L and 10 mmol/L, indicating that a decrease in *MnCTSB-l* expressions leads to the decreased ability to resist alkali treatment in *M. nipponense*. These results collectively indicate that *CTSB-l* positively regulates alkali stress responses in *M. nipponense*.

## 4. Materials and Methods

### 4.1. Experimental Animals and Sample Collection

Healthy *M. nipponense* with body weights of 1.12 ± 0.16 g were provided by the Dapu *M. nipponense* Breeding Base in Wuxi, China (120°13′44″ E, 31°28′22″ N). Prawns were acclimatized for 3 days under controlled conditions: water temperature 28 ± 1 °C, pH 7.81–8.32, dissolved oxygen ≥ 6.0 mg/L, and a 12 h/12 h light–dark cycle. Tissues (eyestalks, cerebral ganglions, hearts, hepatopancreases, gills, gonads) were collected from healthy male and female prawns in order to determine the mRNA expressions in different tissues. Alkali solutions (5 mmol/L and 10 mmol/L) were prepared by dissolving NaHCO_3_ in aerated filtered freshwater, with concentrations verified according to the SC/T9406-2012 standard [49]. Water without alkali was used as the control. A total of 540 prawns were randomly allocated into nine tanks (three tanks per concentration, 60 prawns/tank). Hepatopancreases and gills were collected on days 0, 1, 2, 5, 10, and 15 after alkali treatment. Three tissues (including male and female prawns) were pooled together to form a biological replicate, and six biological replicates were prepared for each time point, and the ratio of male to female prawns for all biological replicates was 1:1. All tissue samples were stored at −80 °C until the RNA extraction.

### 4.2. Total RNA Extraction and Rapid Amplification of cDNA Ends (RACE) of MnCTSB-l Gene

Total RNA was extracted from the hepatopancreas of *M. nipponense* using the RNAiso Plus Kit (TaKaRa, Tokyo, Japan). RNA concentration and purity were measured with a NanoDrop One spectrophotometer (Thermo Scientific, Waltham, MA, USA), and integrity was confirmed via 1.2% agarose gel electrophoresis. First-strand cDNA synthesis was performed using the M-MLV Reverse Transcriptase Kit (TaKaRa, Tokyo, Japan) following the manufacturer’s protocol, with synthesized cDNA stored at −20 °C. The cDNA fragment of *MnCTSB-l* was obtained from the gill transcriptome of *M. nipponense* (NCBI Accession: PRJNA1048646). 3′- and 5′-RACE amplifications were conducted using a SMARTer RACE PCR Kit (TaKaRa, Tokyo, Japan), with primers designed via Primer 5.0 software (Table 1). Experimental procedures followed established methods [50]. PCR products were measured by 1.2% agarose gel electrophoresis and then purified and sequenced by Shanghai Sangon Biotech (Shanghai, China).

### 4.3. Bioinformatics Analysis

The 5′ and 3′ sequences from the RACE processes were assembled with the corresponding partial cDNA fragments using DNAMAN 9.0. The open reading frame (ORF) of *MnCTSB-l* nucleotide sequence was predicted via the ORF Finder (https://www.ncbi.nlm.nih.gov/orffinder/, accessed on 9 October 2024). The molecular weight (MW) and the isoelectric point (PI) of the predicted *MnCTSB-l* amino acid sequence were calculated using the Protparam program in ExPasy (https://web.expasy.org/protparam/, accessed on 11 October 2024). Signal peptide cleavage sites and protein structure–function domains were predicted with SignalP 5.0 (https://services.healthtech.dtu.dk/services/SignalP-5.0/, accessed on 11 October 2024) and InterProScan (http://www.ebi.ac.uk/interpro/, accessed on 11 October 2024). Potential N-glycosylation sites were predicted using the NetNGlyc 1.0 server (http://www.cbs.dtu.dk/services/NetNGlyc/, accessed on 11 October 2024). The BLASTx in NCBI was used to search the similarity of *MnCTSB-l* amino acid sequence with the other *CTSB-l* amino acid sequences (https://blast.ncbi.nlm.nih.gov/Blast.cgi, accessed on 12 October 2024), and multiple sequence alignment was performed by using DNAMAN 9.0 software. A phylogenetic tree was constructed using the neighbor-joining method (bootstrap test of 1000 replications) via MEGA 11 software. The *CTSB-l* amino acid sequences used for the construction of phylogenetic tree are listed in Table 2.

### 4.4. qPCR Analysis

qPCR was performed to measure *MnCTSB-l* mRNA expression. Total RNA extraction and cDNA synthesis followed the protocol described in Section 4.2. qPCR procedures were performed as previously established using the UltraSYBR Mixture (CWBIO, Beijing, China) on a Bio-Rad iCycler iQ5 real-time PCR system (Bio-Rad, Hercules, CA, USA) [51,52]. The PCR thermal cycling profile is 95 °C for 30 s, followed by 40 cycles of 95 °C for 10 s, 55 °C for 10 s, and 72 °C for 30 s. Eukaryotic translation initiation factor 5A (*EIF*) was used as the internal reference gene, which has been proven to be a suitable reference gene for qPCR analysis in *M. nipponense* [51]. The specific primers used for qPCR are listed in Table 1. The relative mRNA expression levels of *MnCTSB-l* were calculated using the 2^−ΔΔCT^ method [53].

### 4.5. In Situ Hybridization (ISH)

Hepatopancreas and gill tissues of healthy *M. nipponense* without alkali treatment were collected from both male and female prawns and were fixed in 4% paraformaldehyde. The anti-sense and sense probes with DIG signal were designed based on the cDNA sequence of *MnCTSB-l* using primer 5.0 software. The sense probe was used as the negative control. ISH protocols followed published methodologies [54], with slides imaged under light microscopy.

### 4.6. RNAi Analysis

RNAi analysis was used to verify the potential functions of *MnCTSB-l* in response to the alkali stress. Specific primers with T7 promoter site were designed using the online software Snap Dragon (https://www.flyrnai.org/cgi-bin/RNAi_find_primers.pl, accessed on 12 October 2024). The double-stranded RNA of *MnCTSB-l* (*dsCTSB*) was synthesized using the Transcript Aid™ T7 High Yield Transcription Kit (Fermentas, Inc., Rockville, MD, USA). The double-stranded RNA of green fluorescent protein (*dsGFP*) was used as a negative control [55]. The *dsRNA* integrity was detected by 1.2% agarose gel electrophoresis, with concentrations quantified using a BioPhotometer (Eppendorf, Hamburg, Germany). The synthesized *dsCTSB* and *dsGFP* were stored at −80 °C. 

Two experimental groups were established: *dsGFP*-injected group (control) and *dsCTSB*-injected group (RNAi). A short-term interference experiment was performed to verify the interference efficiency. A total of 100 healthy prawns were randomly selected for each group, and hepatopancreases were collected at days 0, 1, 4, and 7 after injection. Tissue collection was carried out in the same way as described in Section 4.1, and qPCR was used to determine *MnCTSB-l* expression.

For the long-term interference experiment, 540 healthy prawns were exposed to 5 mmol/L or 10 mmol/L alkali stress, divided equally into *dsGFP*/*dsCTSB* groups (3 replicates/group, *n* = 45/replicate). dsRNA was injected every 5 days to maintain the interference efficiency. Cumulative mortality rates were recorded on days 1, 4, 8, 12, and 16 after injection.

### 4.7. Data Analysis

All data were expressed as means ± SD and were analyzed using SPSS Statistics 25.0 (IBM, Armonk, NY, USA). Statistical differences were determined by independent samples *t*-test and one-way analysis of variance (Duncan’s multiple range test). Differences were considered significant when *p* < 0.05.

## 5. Conclusions

In conclusion, the present study identifies the potential role of *MnCTSB-l* in response to alkali stress in *M. nipponense*. *MnCTSB-l* was expressed in all tested tissues of *M. nipponense*, with the highest expression levels in hepatopancreases of both male and female prawns, suggesting its involvement in immune regulation during aquatic environmental changes. Furthermore, the expressions of *MnCTSB-l* in gills under alkali stress conditions further suggested the potential functions of *CTSB-l* in response to alkali stress in *M. nipponense*. RNAi analysis revealed that the decrease in *MnCTSB-l* expressions resulted in an increase in mortality rate during the alkali treatment, indicating that this gene positively regulates the immune response to changes in alkalinity. The present study provides valuable evidence for the analysis of alkalinity acclimation in *M. nipponense*, thus promoting the genetic improvement of this species.

## Figures and Tables

**Table 1:** Primers used in this study.

Primer Name	Sequence (5′-3′)	Purpose
MnCTSB-l 3GSP1	GATATCATGACTAATGGCCCCGT	3′ RACE
MnCTSB-l 3GSP2	GGCGCCGAAGTCTATAGTTTCTA	3′ RACE
MnCTSB-l 5GSP1	ACTTCAGCAATGGTTGGACAATG	5′ RACE
MnCTSB-l 5GSP2	CTGTGGATACAAGCTCTGTCACT	5′ RACE
MnCTSB-l F1	TAGCAGCGTCACCATTAGGTATC	ORF
MnCTSB-l R1	ACTTCAGCAATGGTTGGACAATG	ORF
MnCTSB-l F2	CATTGTCCAACCATTGCTGAAGT	ORF
MnCTSB-l R2	TCATACTGCATTTCCTCCTCCTG	ORF
MnCTSB-l F3	GGCGCCGAAGTCTATAGTTTCTA	ORF
MnCTSB-l R3	TTCACCACCAGTTAAGCTGGAAT	ORF
MnCTSB-l F	ATTCTCTTACTGGGAAAGGTCCG	qPCR
MnCTSB-l R	GCTGGTATTCGTCTATGCAGACT	qPCR
EIF-F	CATGGATGTACCTGTGGTGAAAC	qPCR
EIF-R	CTGTCAGCAGAAGGTCCTCATTA	qPCR
MnCTSB-l dsF	TAATACGACTCACTATAGGGGTGGATCTTGTTGGGCTGTT	RNAi
MnCTSB-l dsR	TAATACGACTCACTATAGGGCTATGCAGACTTCCGTGCAA	RNAi
GFP dsF	GATCACTAATACGACTCACTATAGGGTCCTGGTCGAGCTGGACGG	RNAi
GFP dsR	GATCACTAATACGACTCACTATAGGGCGCTTCTCGTTGGGGTCTTTG	RNAi
MnCTSB-l antisense-probe	TTCCAGGGGTGTGTCCTCTGCGAAGTTA	ISH
MnCTSB-l sense-probe	TAACTTCGCAGAGGACACACCCCTGGAA	ISH

**Table 2:** Species used for the construction of the phylogenetic tree in the present study.

Species	Accession Number
*Macrobrachium rosenbergii*	XP_066980952.1
*Palaemon carinicauda*	XP_068219370.1
*Adelges cooleyi*	XP_050425328.1
*Macrosteles quadrilineatus*	XP_054283697.1
*Spodoptera frugiperda*	XP_035432705.1
*Cydia strobilella*	XP_063548970.1
*Cydia amplana*	XP_063361903.1
*Zophobas morio*	XP_063919707.1
*Diabrotica virgifera virgifera*	CAE47498.1
*Parasteatoda tepidariorum*	XP_042911940.1
*Spodoptera exigua*	ABK90823.1
*Cydia fagiglandana*	XP_063380485.1
*Homalodisca vitripennis*	XP_046685171.1

## Data Availability

The original contributions presented in the study are included in the article, further inquiries can be directed to the corresponding authors.
